# The quadrivalent HPV vaccine is protective against genital warts: a meta-analysis

**DOI:** 10.1186/s12889-020-08753-y

**Published:** 2020-05-28

**Authors:** Anita Lukács, Zsuzsanna Máté, Nelli Farkas, Alexandra Mikó, Judit Tenk, Péter Hegyi, Balázs Németh, László Márk Czumbel, Sadaeng Wuttapon, István Kiss, Zoltán Gyöngyi, Gábor Varga, Zoltán Rumbus, Andrea Szabó

**Affiliations:** 1 Department of Public Health, Faculty of Medicine, University of Szeged, Dóm tér 10, Szeged, 6720 Hungary; 2 Institute for Translational Medicine, Medical School, University of Pécs, Pécs, Hungary; 3 Department of Public Health Medicine, Medical School, University of Pécs, Pécs, Hungary; 4 Department of Oral Biology, Faculty of Dentistry, Semmelweis University, Budapest, Hungary

**Keywords:** Human papillomavirus, Vaccination, Prevention, Genital wart

## Abstract

**Background:**

The quadrivalent human papillomavirus (HPV) vaccine has been assumed to give protection against genital warts (GW) as well as cervical cancer. Our main question was whether HPV vaccine has any effects on the prevention of GW reported in randomised controlled clinical trials (RCTs) and time-trend analyses.

**Methods:**

This meta-analysis was performed according to the PRISMA guidelines using the PICO format. We searched in three electronic databases (PubMed, Embase, Cochrane Trials), and assessed heterogeneity using the Q-test and I-squared statistics, meta-regression was also performed. Odds ratios (OR) and their confidence intervals (CI) were calculated. The sensitivity was tested by leave-one-out method. We evaluated the presence of publication bias using the funnel plot graph and the Copas selection model. The strength of evidence was assessed using the GRADE approach.

**Results:**

Eight RCTs (per-protocol populations) and eight time-trend ecological studies were included in this meta-analysis. A significant reduction (pooled OR = 0.03, 95% CI: 0.01–0.09; I-squared = 53.6%) of GW in young women was recorded in RCTs, and in time-trend analyses both in young women (pooled OR = 0.36, CI 95% = 0.26–0.51; I-squared = 98.2%), and in young men (pooled OR = 0.69, 95% CI = 0.61–0.78; I-squared = 92.7%). In subgroup analysis, a significant reduction of the number of GW events was observed especially in women under 21 years (pooled OR = 0.33, 95% CI = 0.17–0.63). Leave-one-out analysis showed that similar results could be obtained after excluding one study, meta-regression did not show significant difference.

**Conclusions:**

Prophylactic, quadrivalent HPV vaccination can prevent GW in healthy women and men, therefore, it should be included in routine immunization programme.

## Background

Human papillomaviruses (HPV) are sexually transmitted infectious agents and are increasingly common in young people. Three HPV vaccines are currently available worldwide which cover 2, 4, or 9 HPV genotypes [1]. The bivalent vaccine (Cervarix®; GlaxoSmithKline) targets HPV types 16 and 18, which are associated with 70–80% of cervical cancers globally. Besides these two genotypes, the quadrivalent vaccine (Gardasil®; Merck & Co) – which was licensed by the US Food and Drug Administration (FDA) in 2006 – also targets HPV types 6 and 11, associated with 85–95% of cases of genital warts (GW). Recently a nine-valent (HPV 6/11/16/18/31/33/45/52/58) HPV vaccine (Gardasil®9; Merck Sharp & Dohme Corp) has been licensed, which provides high and consistent protection against infections and diseases related to these HPV types [2].

The World Health Organization (WHO) recognizes the importance of cervical cancer and other HPV-related diseases as global public health problems and reiterates the recommendation that HPV vaccines should be included in national immunization programmes [3]. WHO recommends vaccination for girls aged 9–13 years as this is the most cost-effective public health measure against cervical cancer [4]. By October 2019, 98 countries introduced HPV vaccines through national immunisation programmes [5]. The population-level effect of HPV vaccination programmes is expected to vary substantially between these countries, depending on the vaccine used, implementation strategies applied and vaccination coverage achieved [6].

As the quadrivalent vaccine became available in 2006, HPV vaccination has been introduced in an increasing number of high-income countries, while in low- and middle-income countries the bivalent, or both vaccines are funded. There is high certainty evidence that HPV vaccines protect against cervical precancer (cervical intraepithelial neoplasia grade 2/3 and above) in adolescent girls and young women who are vaccinated between aged 15 to 26 [7], and growing evidence support that introduction of HPV vaccines reduces HPV related diseases, including precancerous or dysplastic lesions [8–11]. By achieving high vaccination coverage (> 80%) in girls, the risk of HPV infection for boys can also be reduced and its elimination could also be achieved [12]. Moreover, the quadrivalent vaccine has also been found to be highly effective in preventing genital lesions associated with infection with HPV 6, 11, 16, or 18 in men [13]. Therefore, several countries, such as the United States of America (USA), Australia, Switzerland, Austria, and Canada, have started to vaccinate boys as well as the quadrivalent vaccination prevents genital cancers and GW in both males and females.

Although GW are usually not referred to be a serious condition, the loss of quality of life of patients, physical symptoms such as pruritus and pain, and the impact on sexuality can be addressed as significant issues [14].

In the present work our aim was to summarize the available evidence on the efficacy of the quadrivalent HPV vaccine in preventing GW by conducting a meta-analysis. To achieve this, the following PICO (patients, intervention, comparison, outcome) format was applied: P: human population; I: quadrivalent HPV vaccine; C: no vaccine/placebo and O: reduced incidence/prevalence of GW.

## Methods

The present meta-analysis was performed using the Preferred Reporting Items for Systematic Review and Meta-Analyses protocols (PRISMA protocol) [15]. This meta-analysis protocol was registered with the International Prospective Register of Systematic Reviews (PROSPERO), registration number: CRD42018095030.

### Search strategy and selection criteria

We conducted a systematic search in three electronic databases from inception up to 13th of January, 2020, without language restrictions. The following search queries were used: (“genital wart”[All Fields] OR “anogenital wart”[All Fields] OR (“condylomata acuminata”[MeSH Terms] OR (“condylomata”[All Fields] AND “acuminata”[All Fields]) OR “condylomata acuminata”[All Fields] OR “condyloma”[All Fields]) OR “condyloma acuminatum”[All Fields]) AND (“HPV vaccine”[All Fields] OR “HPV vaccination”[All Fields] OR “human papillomavirus vaccine”[All Fields] OR “human papillomavirus vaccination”[All Fields] OR (“human papillomavirus recombinant vaccine quadrivalent, types 6,11,16,18”[MeSH Terms] OR “gardasil”[All Fields])) AND “humans”[MeSH Terms] for PubMed; (“genital wart” OR “anogenital wart” OR condyloma OR “condyloma acuminatum”) AND (“hpv vaccine” OR “hpv vaccination” OR “human papillomavirus vaccine” OR “human papillomavirus vaccination” OR “gardasil”) AND [humans]/lim AND ([article]/lim OR [article in press]/lim OR [conference abstract]/lim OR [conference paper]/lim OR [conference review]/lim OR [review]/lim) for Embase and (“genital wart” OR “anogenital wart” OR condyloma OR “condyloma acuminatum”) AND (“HPV vaccine” OR “HPV vaccination” OR “human papillomavirus vaccine” OR “human papillomavirus vaccination” OR Gardasil) for Cochrane Trials. We identified relevant studies by reviewing titles and abstracts, and the reference lists of relevant articles were also searched to reveal all relevant studies.

We included RCTs and time-trend ecological studies which assessed the effectiveness of the quadrivalent HPV vaccine in the frequency (incidence/prevalence) of GW. In the RCTs, HPV vaccines were administered only to females, except one RCT, where Japanese males were received HPV vaccine or placebo [16]. Similarly to most of included RCTs, only women were vaccinated in ecological studies, but the whole population were included – because the researchers examined the indirect effect of the vaccine on men as well – except in one study [17], where only females were involved. Articles were eligible for inclusion if they provided data about the incidence of GW in the vaccinated and placebo group (RCT studies) or if they assessed the population-level effect of the quadrivalent HPV vaccination by comparing the prevalence of GW between the pre- and post-vaccination periods (time-trend ecological studies).

We excluded studies with the following characteristics: just the bivalent vaccine was used; or bivalent and quadrivalent vaccines were used alternatively; or the quadrivalent HPV vaccination was administered in the framework of private care; or the frequency of GW was studied during the post-vaccination period and the comparison of pre- and post-vaccination period was not included. The present analysis included only articles where the absolute number of GW is available. Narrative reviews, mathematical modelling studies, conference abstracts, and conference papers were also excluded.

### Study selection

The EndNote X7.4 software package was used for record management. After removing duplicates, the remaining records were screened for eligibility by two authors (AL, AS) based on the title and abstract of the published original papers. The eligibility of full texts of the remaining records was assessed by two reviewers independently. Disagreement between reviewers was resolved by discussion or, if it was necessary, by consulting with a third reviewer (ZSM).

### Risk of bias assessment

The quality of the studies was assessed independently by two authors (AL, AS). We carried out a risk of bias assessment of RCTs using tools proposed by Cochrane for RCTs [18]. This tool assesses the risk of bias based on five domains: selection bias, performance bias, detection bias, attrition bias and reporting bias. For ecological studies the risk of bias was assessed using the method of Drolet and his colleagues [6]. We evaluated the risk of bias using three domains: selection bias, information bias, and risk of confounding.

### Data extraction

Data extraction was conducted independently by two investigators (AL, AS). In case of disagreement concerning the inclusion of studies and extracted data, a third investigator (ZSM) was involved to resolve discrepancies.

In RCTs, the data for primary analysis were collected from a per-protocol susceptible population of women, who received all three doses of vaccination and who had no virological evidence of the relevant HPV types through 7 months after administration of the third dose. In RCTs, we collected information about the number of GW in the vaccinated and placebo groups. Afterwards the number of countries included in the given RCT, the length of the follow-up period, the age of the participants and the lifetime number of their sexual partners were also extracted.

In the included ecological trials our primary outcome was the number of GW in the pre- and post-vaccination periods. Secondly, we collected information about the introduction year of the vaccine in the certain country, some details about the vaccination programme, the demographic characteristics of the participants and the duration of the pre- and post-vaccination periods.

### Data analysis

For data synthesis we used the methods recommended by the working group of the Cochrane Collaboration. In the meta-analysis, odds ratios (ORs) as well as their 95% confidence intervals (CI) were calculated from the original data of the articles and were visualized in forest-plots. Subgroup-analysis was done within ecological studies according to gender and according to age groups (younger or older than 21). In case of gender we applied meta-regression as well. Depending on data quality, the random effect model by DerSimonian and Laird [19] was used. The heterogeneity was tested also with Q-test statistic and I-squared. A leave-one-out sensitivity analysis was performed by iteratively removing one study at a time to confirm that our findings were not driven by any single study. Statistical analyses were carried out using the STATA software version 15.0.

### Assessment of publication bias

We evaluated the presence of publication bias using the funnel plot graph. We also performed Copas selection model to test the publication bias, calculations were performed with R (V. 3.5.2) package metasens (V. 0.4–0).

### Assessment of quality of evidence

Strength of the overall body of evidence was assessed using the Grading of Recommendations, Assessment, Development and Evaluation (GRADE) approach. Quality of a body of evidence involves consideration of risk of bias, heterogeneity, directness of evidence, precision of effect and risk of publication bias, as described in Section 12.2.2 of the Cochrane Handbook for Systematic Reviews of Interventions [18]. The quality of evidence was judged as “high”, “moderate”, “low”, or “very low”.

## Results

### Selection of studies

Literature searches were performed in PubMed, Embase, and Cochrane Library, where altogether 1862 references were identified. After removing duplicates, and reading the titles and abstracts, a total of 106 potentially relevant articles remained. Finally, 16 studies (8 RCTs and 8 time-trend analyses) were eligible for inclusion in the meta-analysis with more than 13,000000 participants. All selected studies corresponded only to the quadrivalent vaccine.

**Fig. 1:**
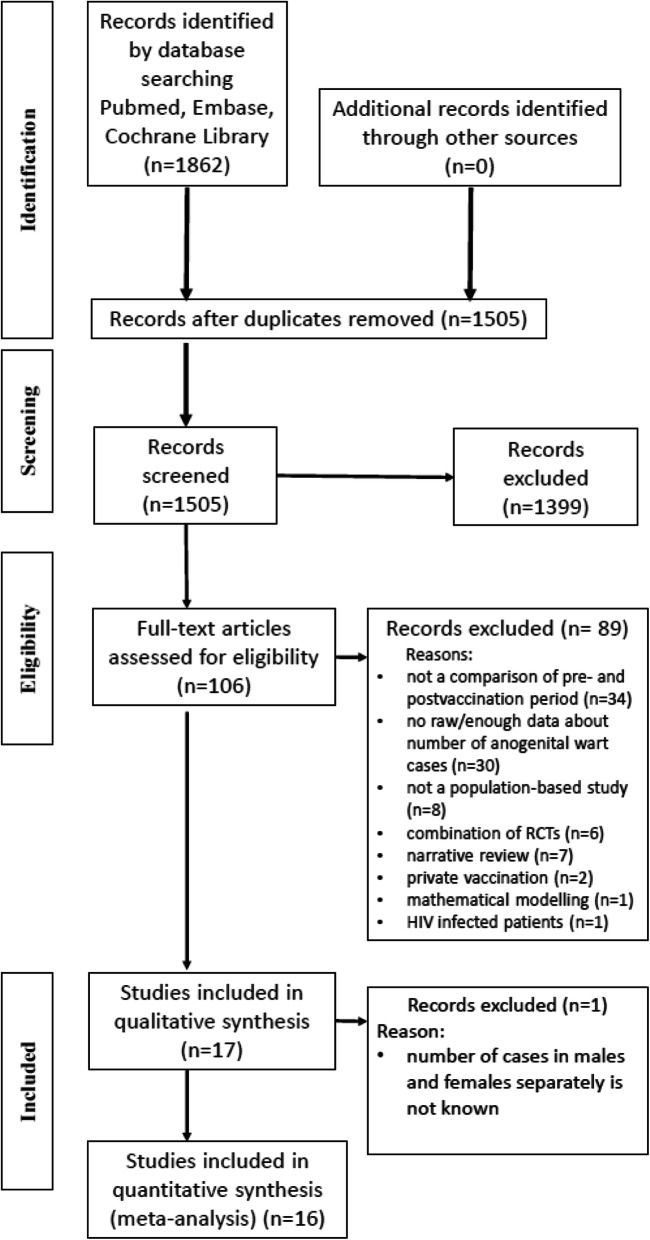
PRISMA 2009 flow diagram for identification of relevant studies

The flow chart for identification of relevant studies and the reasons for exclusions are included in Fig. 1.

### Characteristics of the included studies

Altogether 8 randomised, double-blind, placebo-controlled studies were included in our final quantitative analysis [16, 20–26]. Healthy, non-pregnant women aged between 15 and 26 years with no history of abnormal Pap smear at enrolment were included in seven RCTs, who reported no more than 4 lifetime sexual partners except the Finnish women in Munoz’s study [24] where this restriction was not applied. The total number of enrolled women was 20,416 in the vaccinated group and 20,279 in the placebo group. In the Japanese RCT [16] 561 males were in the vaccinated group and 562 males were received placebo. The mean age of the participants is shown in Table 1. Participants were vaccinated either with the quadrivalent HPV (types 6, 11, 16 and 18) vaccine (vaccinated group) or with placebo (aluminium hydroxyphosphate sulphate adjuvant) by three doses of intramuscular injection. The vaccine was composed of 20 μg of HPV 6, 40 μg of HPV 11, 40 μg of HPV 16 and 20 μg of HPV 18, formulated with 225 μg of aluminium adjuvant. The clinical follow-up periods were between 23 months and 60 months in RCTs. We included the per-protocol population who received all three doses of vaccine or placebo and were seronegative and PCR negative for the relevant HPV types at day 1.

**Table 1:** Characteristics of the studies (RCTs) included in the meta-analysis

First author (year)	Study type	Countries	Average follow-up period	Patient characteristics				Number of cases/number of patients	Number of cases/number of patients
				Age (years)			Number of sexual partners		
				Mean		Range			
				Vaccinated group^**a**^	Placebo group^**b**^			Vaccinated group	Placebo group
Garland (2007) [20]	RCT	16 countries from Asia Pacific, Europe, North- Central- and South America	3 years	20.2 ± 1.8	20.3 ± 1.8	16–24	< 4	0/2261	48/2279
Dillner (2010) [21]	RCT	24 countries from North America, Latin America, Europe, Asia Pacific	42 months	20		16–26	< 4	2/6718	186/6647
Majewski (2009) [22]	RCT	Austria, Czech Republic, Denmark, England, Finland, France, Germany, Greece, Iceland, Ireland, Italy, Norway, Poland, Portugal, Russian Federation, Spain, and Sweden.	36 months	19.7		16–24	≤4	1/4059	90/4057
Villa (2006) [23]	RCT	Brazil, Nordic countries, Finland, Sweden, Norway	60 months	20.2	20.0	16–23	≤4	0/214	20/209
Munoz (2010) [24]	RCT	Australia, Austria, Brazil, Canada, Colombia, Czech Republic, Denmark, Finland, Germany, Hong Kong, Iceland, Italy, Mexico, New Zealand, Norway, Peru, Poland, Puerto Rico, Russia, Singapore, Sweden, Thailand, the United Kingdom, United States	3,6 years	?	?	15–26	< 4 except Finnish women	4/4689	138/4735
Perez (2008) [25]	RCT	Brazil, Mexico, Colombia, Costa Rica, Guatemala, Peru	?	19.8 ± 3.0	20.3 ± 2.2	9–24	≤4	0/2075	9/1976
Yoshikawa (2013) [26]	RCT	Japan	23 months	22.7 ± 2.1	22.9 ± 2.1	18–26	≤4	2/400	7/376
Mikamo (2019) [16]	RCT	Japan	36 months	22.6 ± 2.1	22.6 ± 2.0	18–27	17–26	0/561	1/562

*RCT:* randomised controlled trial

^a^: vaccine = quadrivalent HPV vaccine (HPV types 6, 11, 16, 18)

^b^: placebo = aluminium hydroxyphosphate sulphate adjuvant

Additionally, 8 time-trend analyses [17, 27–33] were included in the present meta-analysis. Five studies [27–31] were performed in Australia, one [17] in Belgium, one [32] in England, and one [33] in the USA (Table 2). The Belgian studies assessed the population-level effect of the quadrivalent HPV vaccination on GW in women [17] and the other 7 articles [27–33] measured the number of GW cases also in men to investigate the herd effect of the vaccine. The quadrivalent vaccine was introduced as a school-based programme in Australia, in 2007. In Belgium, HPV vaccines were reimbursed for women aged 12 to 18 years since 2007. In England, HPV vaccination programme was introduced in 2008, since 2012 the quadrivalent vaccine has been used for females. In the USA, HPV vaccination has been recommended both for girls and boys aged 11–12 years with catch-up vaccination through age 26 years. The investigators compared the number of GW between the pre- and post-vaccination periods and they evaluated the real-life benefit of the introduction of the quadrivalent HPV vaccine on GW at population level.

**Table 2:** Characteristics of the studies (time-trend analysis) included in the meta-analysis

First author (year)	Study type	Country	Year of vaccine introduction	Programme description	Pre-vaccination period	Post-vaccination period	Patient characteristics age group + gender	Number of patients diagnosed with GW/ overall population	
								Pre-vaccination period	Post-vaccination period
Dominiak-Felden (2015) [17]	time-trend analysis	Belgium	2007	Reimbursed for: • women 12 to 18 years old	2006–2007	2007–2013	16–22 years women	244/63180	12/24791
Chow (2015) [28]	time-trend analysis	Australia	2007	School-based programme for: • girls 12–13 years Catch-up programmes (2007–2009) for: • 13–18 years old schoolgirls • 18–26 years old women	2004–2007	2007–2014	Australian-born women < 21 years	159/787	74/1340
							Australian-born women 21–32 years	378/2801	322/5662
							Australian-born heterosexual men < 21 years	62/531	112/1531
							Australian-born heterosexual men 21–32 years	520/2726	789/6539
Ali (2013) [27]	time-trend analysis	Australia	2007	School-based programme for: • girls 12–13 years Catch-up programmes (2007–2009) for: • 13–18 years old schoolgirls • 18–26 years old women	2004–2007	2007–2011	< 21 years women	405/3949	136/5456
							21–30 years women	942/7683	407/7545
							< 21 years heterosexual men	132/1289	93/2693
							21–30 years heterosexual men	1195/6617	1034/8530
Harrison (2014) [29]	time-trend analysis	Australia	2007	School-based programme for: • girls 12–13 years Catch-up programmes (2007–2009) for: • 13–18 years old schoolgirls • 18–26 years old women	2002–2006	2008–2012	15–27 years women	189/43596	71/42393
							15–27 years men	103/21157	87/18745
Read (2011) [31]	time-trend analysis	Australia	2007	School-based programme for: • girls 12–13 years Catch-up programmes (2007–2009) for: • 13–18 years old schoolgirls • 18–26 years old women	2004–2007	2007–2011	< 21 years women	168/886	70/898
							21–29 years women	371/2808	247/3546
							< 21 years heterosexual men	53/378	45/445
							21–29 years heterosexual men	460/2524	500/3479
Fairley (2009) [30]	time-trend analysis	Australia	2007	School-based programme for: • girls 12–13 years Catch-up programmes (2007–2009 for: • 13–18 years old schoolgirls • 18–26 years old women	2004–2007	2008	< 28 years women	850/6693	130/1970
							all men	2024/16727	473/4778
Checchi (2019) [32]	time-trend analysis	England	2012	School-based programme for: • girls 12–13 years Catch-up programmes for: • all females up to 18 years	2014	2017	15–24 years females	18,973/ 3,341,260	13,170/ 3,282,554
							15–24 years heterosexual males	15,981/ 3,395,435	11,601/ 3,356,744
Mann (2019) [33]	time-trend analysis	USA	2011	School-based programme for: • girls 11–12 years • boys 11–12 years Catch-up programmes for: • girls up to 26 years • boys up to 21 years (for bisexual, MSM up to 26 years)	2010	2016	all females (median age: 26 years)	973/42289	193/21484
							all males (median age: 29 years)	3584/49097	1187/26983

*GW:* genital wart, *MSM:* men who have sex with men

### Effectiveness of quadrivalent HPV vaccine on GW

**Fig. 2:**
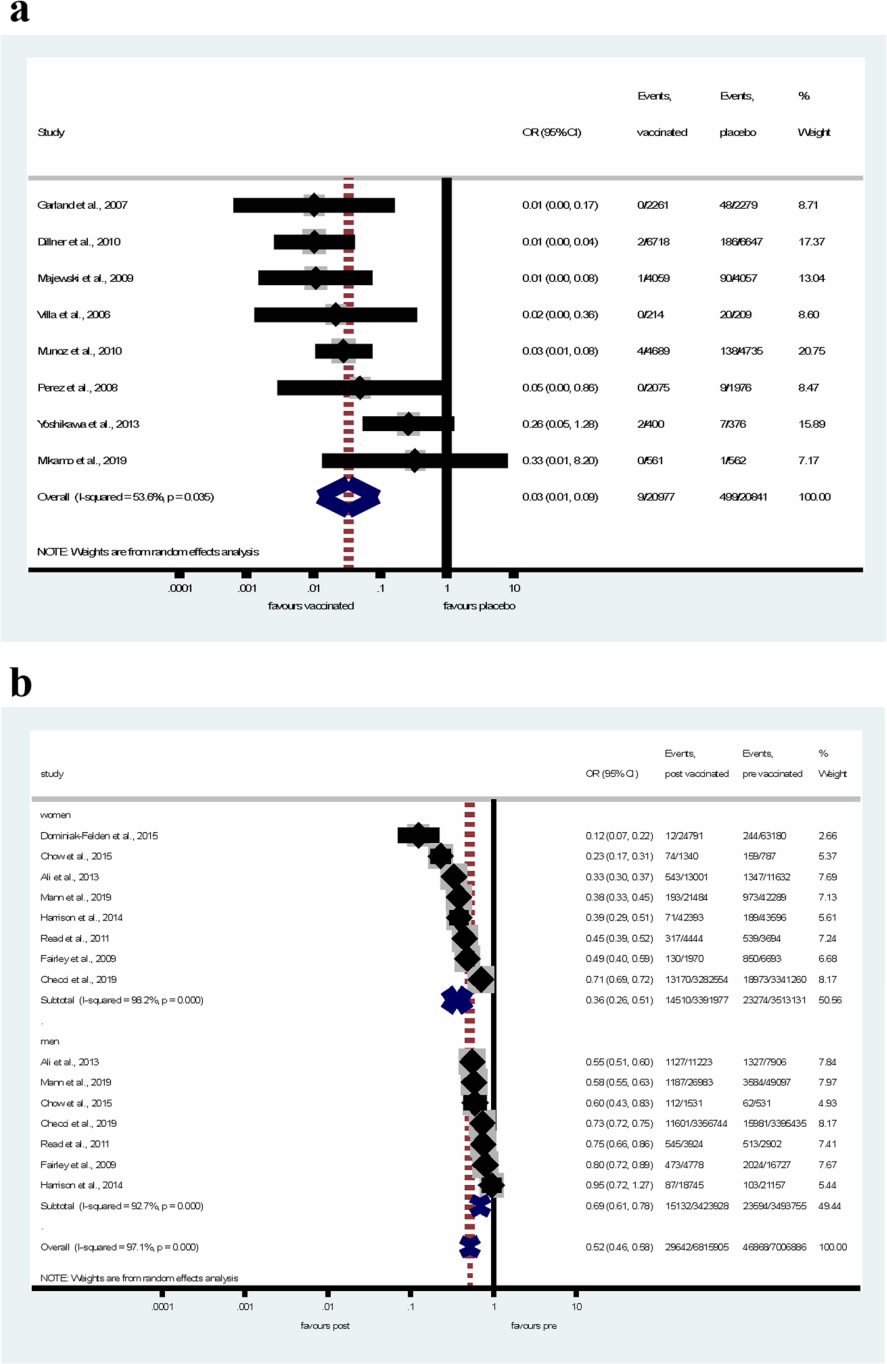
Forest plot of the effectiveness of HPV quadrivalent vaccine in the prevention of GW in RCTs (**a**) and ecological studies (**b**)

According to the results of RCTs, the quadrivalent HPV vaccine significantly reduced the risk of GW (pooled OR = 0.03, 95% CI: 0.01–0.09) in healthy women as shown in Fig. 2a. Although high heterogeneity was reported across studies (I-squared = 53.6%, *p* = 0.035).

In time-trend analyses (Fig. 2b), the number of GW events decreased significantly, by 64% (pooled OR = 0.36, CI 95% = 0.26–0.51) in the post-vaccination period in women. In young men, despite the fact, that they were not vaccinated, the reduction in GW cases was also significant (pooled OR = 0.69, 95% CI = 0.61–0.78), although not as prominent as seen in women. The overall population effect of quadrivalent vaccine was protective against GW (pooled OR = 0.52, 95% CI = 0.46–0.58). The heterogeneity was considerable both in articles involving women (I-squared = 98.2%, *p* < 0.001) and men (I-squared = 92.7%, p < 0.001).

**Fig. 3:**
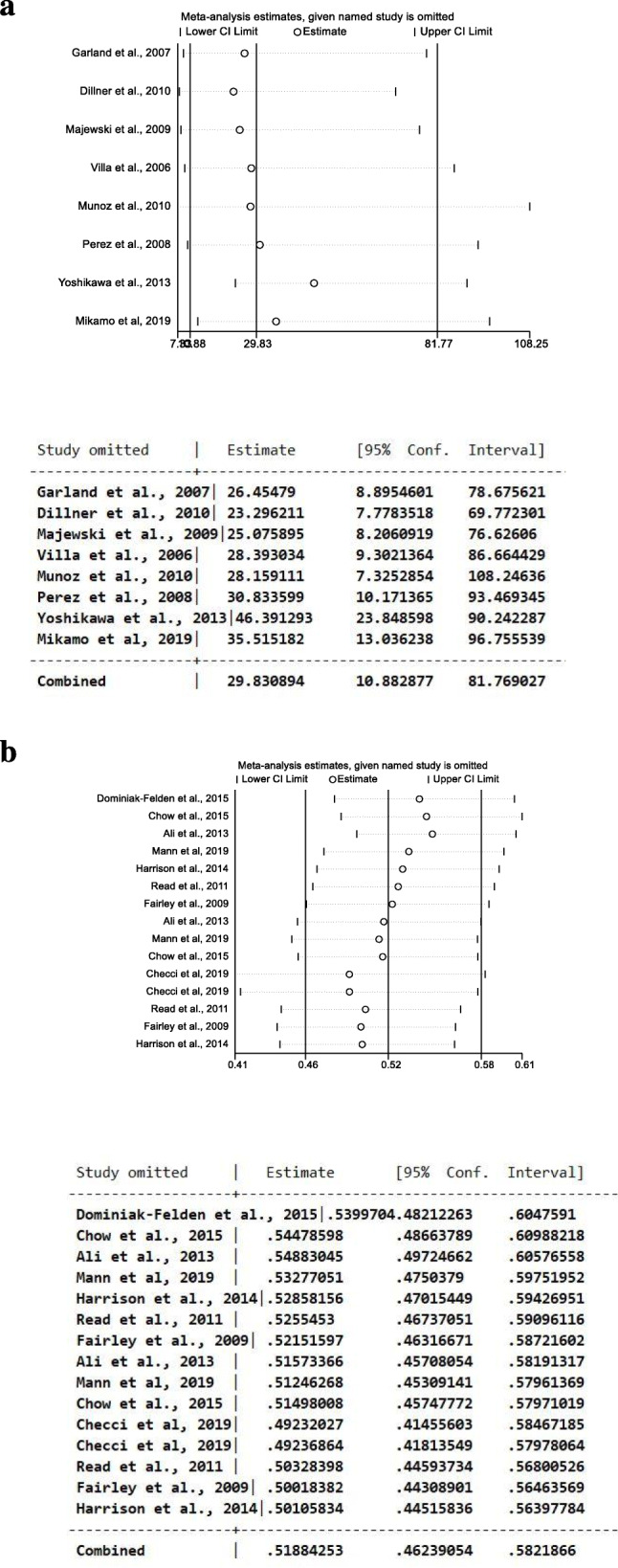
Results of leave-one-out sensitivity analysis (plot and numbers) in RCTs (**a**) and in ecological studies (**b**) The vertical axis shows the omitted study. The horizontal axis represents the odds ratio. Every circle indicates the pooled OR when the left study is omitted in this meta-analysis. The two ends of every broken line represent the respective 95% confidence interval (CI)

We performed a leave-one-out sensitivity analysis by iteratively removing one study at a time and recalculating the summary OR. The summary ORs remained stable both in RCTs (Fig. 3a) and in ecological studies (Fig. 3b), indicating that our results were not driven by any single study and that similar results could be obtained after excluding one study.

**Fig. 4:**
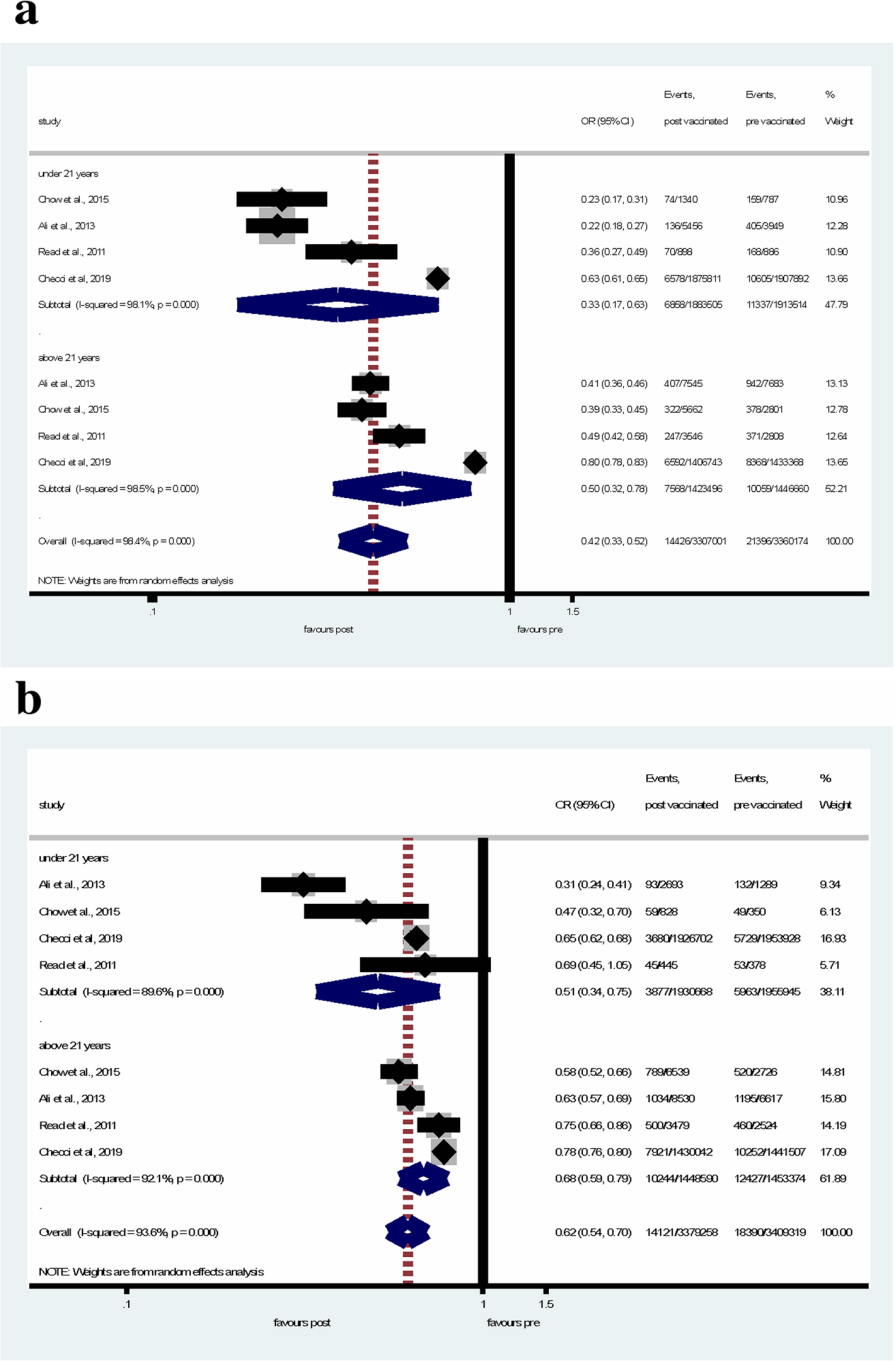
Subgroup analyses of the changes in GW diagnosis between the pre- and post-vaccination periods in women (**a**) under 21 years of age and over 21 years of age, and in men (**b**) under 21 years of age and over 21 years of age

In subgroup analysis, when the age-groups were separated at 21 years, in women under 21 years of age, the number of GW events decreased significantly by 67% (pooled OR = 0.33, 95% CI = 0.17–0.63), in women over 21 years of age, GW reduced less substantially (by 50%) (pooled OR = 0.50, 95% CI = 0.32–0.78) (Fig. 4a). In men under 21 years of age, GW reduced significantly by 49% (pooled OR = 0.51, 95% CI = 0.34–0.75) in the post-vaccination period, the reduction of GW was also significant in men over 21 years old (pooled OR = 0.68, 95% CI = 0.59–0.79) (Fig. 4b).

**Fig. 5:**
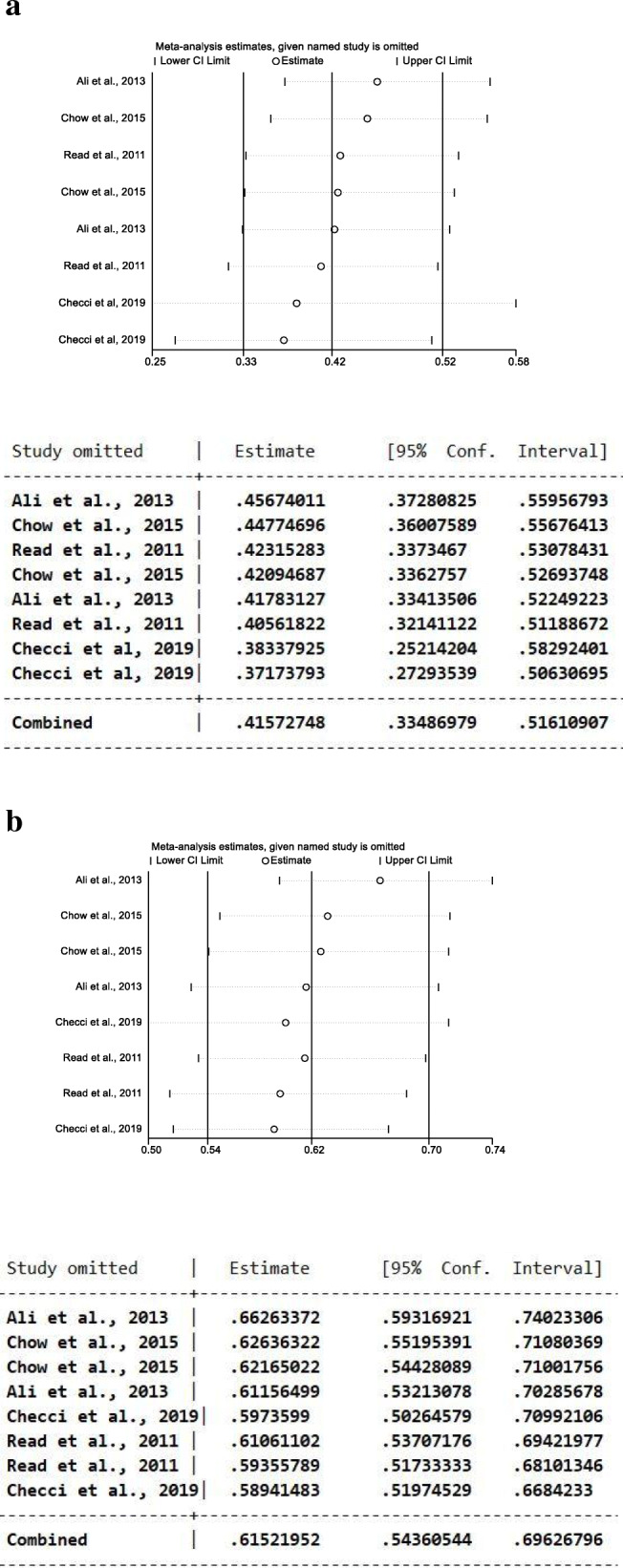
Results of leave-one-out sensitivity analysis (plot and numbers) among studies in women (**a**) and in men (**b**). The vertical axis shows the omitted study. The horizontal axis represents the odds ratio. Every circle indicates the pooled OR when the left study is omitted in this meta-analysis. The two ends of every broken line represent the respective 95% confidence interval (CI)

Based on the results of leave-one-out sensitivity analysis (Fig. 5a, b), removing one study showed similar and consistent result.

**Fig. 6:**
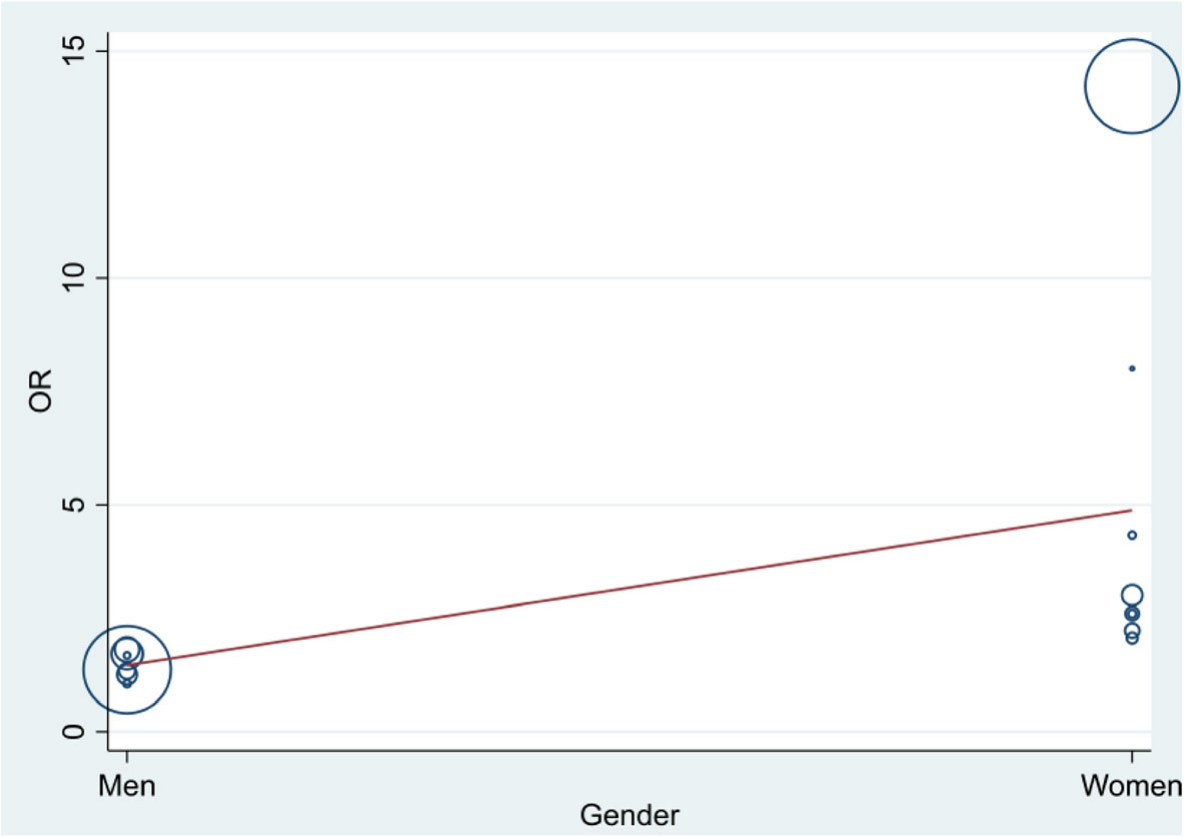
“Bubble plot” with fitted meta-regression line. OR = odds ratio

Meta-regression also did not show significant difference (*p* = 0.54) among odds ratios of studies (Fig. 6).

### Publication bias

The results of Copas selection model are included as Additional file 1. No publication bias was observed in the analysis of RCTs (**A**). In the analysis of ecological studies (**B**) publication bias may occur, but the leave-one-out analysis did not show inconsistent result.

### Risk of bias assessment

**Fig. 7:**
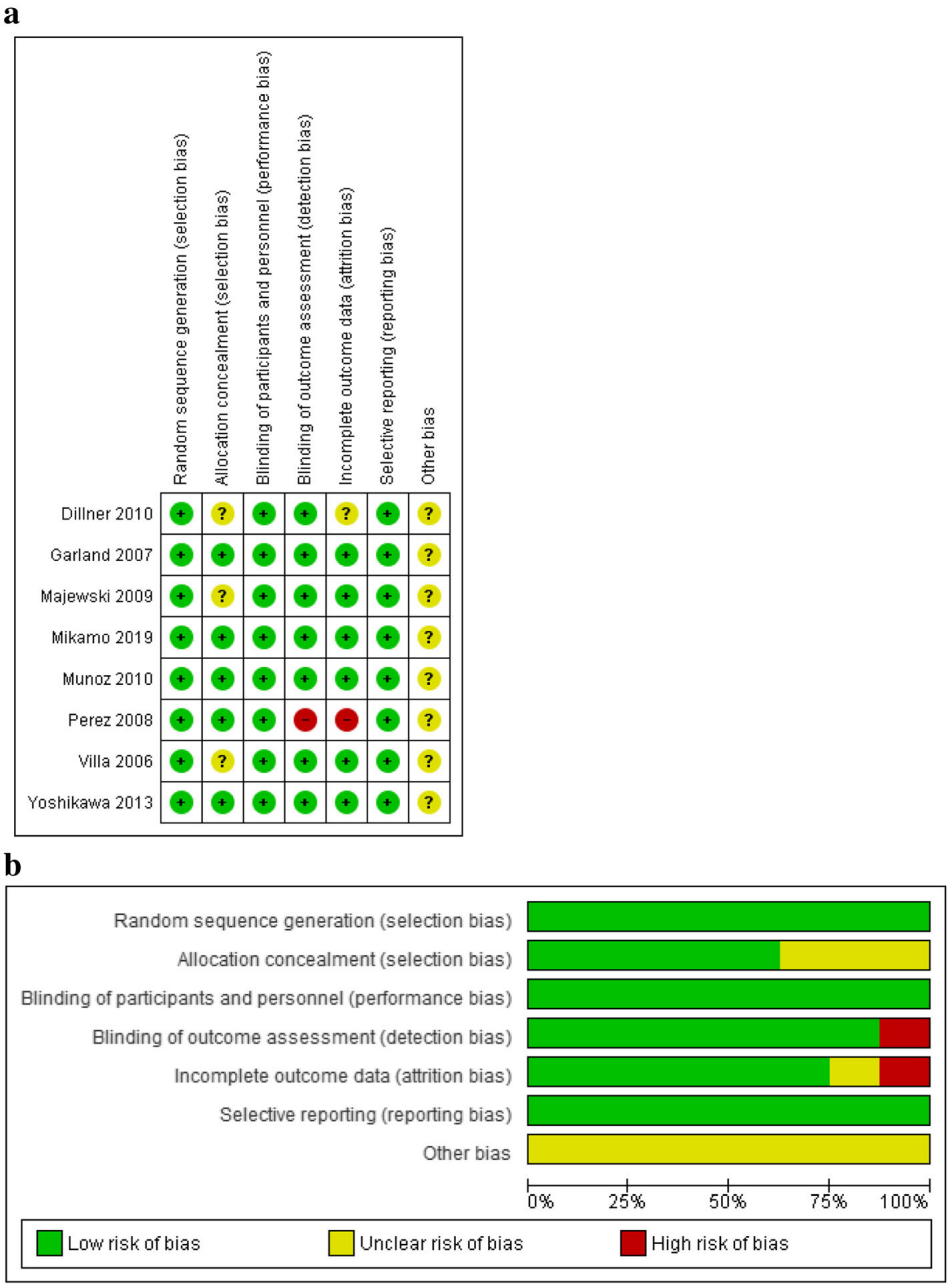
Risk of bias summary: review of authors’ judgement on each risk of bias item for each included RCT study (**a**); and risk of bias graph: review of authors’ judgement on each risk of bias item, presented as percentages across all included RCT studies (**b**)

Among RCTs only one study [25] was rated as high risk of detection and attrition bias due to the lack of blinded investigation of the samples by pathologists and also to missing outcome data (Fig. 7). The randomization, performance and reporting bias were considered low in the case of all studies. 3 RCTs [21–23] showed unclear selection bias and in article Dillner and co-workers [21] some data might not have been revealed and published (unclear attrition bias).

Most of the time-trend analyses showed high risk of confounding factors such as changes in sexual activity and health-seeking behaviour during the long period of the study, which can potentially cause changes in GW. The majority of studies showed low risk of information bias because GW were diagnosed by physicians except in the study of Dominiak-Felden [17], where a surrogate marker was used as definition of cases. Due to the possible changes of clientele of sexual health services between the pre- and post-vaccination periods, the risk of selection bias was evaluated as unclear or high. Moreover, in two studies [27, 28] the vaccination status and the number of doses of HPV vaccines were self-reported, therefore the risk of selection bias was high (Table 3).

**Table 3:** Risk of bias in time-trend analyses

First author (year)	Risk of selection bias: changes in the study population characteristics between the pre- and post-vaccination periods	Risk of information bias: errors in the identification of HPV+ during the pre- and post-vaccination period (data source, genital wart case definition, outcome used)	Risk of confounding: changes in HPV infection between the pre- and post-vaccination periods could be diluted/exacerbated by other variables
Dominiak-Felden (2015) [17]	**High** Some people possibly may have been vaccinated without reimbursement (risk of misclassification), imiquimod agreement was used as the date of vaccination.	**High** A surrogate marker (imiquimod agreement) was used as definition of genital wart cases (risk of underestimation).	**High** Different sexual behaviours between vaccinated and unvaccinated women.
Chow (2015) [28]	**High** Possible changes in the clientele of the sexual health services between the periods. Self-reported vaccination status and the number of doses of HPV vaccine.	**Low** Clinical diagnosis by clinicians.	**High** Clients at sexual health service have higher risk of sexually transmissible infections.
Ali (2013) [27]	**High** Possible changes in the clientele of the sexual health services between the periods. Self-reported vaccination status and the number of doses of HPV vaccine.	**Low** Genital warts are directly diagnosed by physicians.	**High** Changes in sexual activity, health seeking behaviour could potentially cause changes in genital wart frequency over time.
Harrison (2014) [29]	**Unclear** Some women from the vaccination eligible group may be included with non-vaccine eligible women due to the change in patient age.	**Low** Genital wart diagnosis by physicians.	**High** Change in sexual risk behaviour.
Read (2011) [31]	**Unclear** Possible changes in the clientele of the Melbourne Sexual Health Centre.	**Low** Genital wart diagnosis by physicians.	**Unclear** Possible HPV infection of 21–29 years women before the vaccination.
Fairley (2009) [30]	**Unclear** Possible changes in the clientele of the Melbourne Sexual Health Centre.	**Low** Genital wart diagnosis by physicians.	**Unclear** Boys aged 9–15 years could be prescribed the vaccine privately.
Checchi (2019) [32]	**Unclear** Inability to link anogenital wart diagnoses to individual vaccination status.	**Low** Genital wart diagnosis by physicians.	**Unclear** Patients could attend elsewhere for treatment of anogenital wart.
Mann (2019) [33]	**Unclear** Patients’ vaccination status is unknown.	**Low** Genital wart diagnosis by physicians.	**Unclear** Patients with anogenital wart could choose to seek care elsewhere.

### Level of evidence assessment

The GRADE evidence profiles are shown in Table 4. By their very nature, RCTs are considered “high quality” but we downgraded them by 1 because the per-protocol population in RCTs does not represent the whole population due to the strict inclusion and exclusion criteria. As a consequence, the GRADE level of evidence can be regarded as moderate for RCTs. The natural “low quality” of time-trend analyses was first down- than upgraded, resulting in “low quality” according to GRADE.

**Table 4:** Rating the quality of evidence using the GRADE methodology

Num-ber of studies	Study design	Initial level of evi-dence	Evidence components	Downgrade/upgrade of evidence	Notes	Final level of evidence
**8**	**RCT**	**high**	Risk of bias	–	**–**	**⨁⨁⨁O** **MODERATE**
			Heterogeneity	–	**–**	
			Indirectness	downgrade by 1	groups with different background risks	
			Imprecision	–	**–**	
			Publication bias	–	**–**	
**8**	**time-trend analysis**	**low**	Risk of bias	–	–	**⨁⨁OO** **LOW**
			Heterogeneity	downgrade by 1	low *p* value	
			Indirectness	–	**–**	
			Imprecision	upgrade by 1	high number of participants, narrow CI	
			Publication bias	–	**–**	

## Discussion

In the present study, we investigated the incidence of GW in healthy women after the administration of quadrivalent HPV vaccination (or placebo) and compared the presence of GW in women and men before and after the administration of the quadrivalent HPV vaccine. Altogether 16 studies (including 8 RCTs) were included in the current meta-analysis. We found that the quadrivalent HPV vaccine is effective in preventing GW in women. According to the results of subgroup analyses, GW reduction was more substantially in women under 21 years of age. Furthermore, a significant herd protection was observed in young men.

Leave-one-out analysis showed that similar results could be obtained after excluding one study.

RCTs have clearly demonstrated the efficacy of the quadrivalent HPV vaccine in vaccinated persons but not investigated the effects at population level and in men. At population-level, a reduction in the prevalence of GW was reported due to HPV vaccination. In men, we also observed a significant decline in GW, which can most likely be attributed to the indirect protection provided by the vaccination of women by the quadrivalent vaccine. This so called “herd immunity” is the protective effect of the vaccine extending beyond the vaccinated individuals to the unvaccinated ones in the population [34].

Overall, most of the RCTs showed low or unclear risk of bias, therefore, the methodological quality of the trials is acceptable. Ecological studies provide valuable timely information about the population-level effects of HPV vaccination using large populations. However, these studies are vulnerable to carry risk of bias. The possible changes in the clientele and/or in sexual activity or health-seeking behaviour of the clients can modify the diagnosis of GW over time. Despite the unclear/high risk of selection bias and confounding factors, it was important to use the results of time-trend analyses to evaluate the performance of the vaccine under real-life settings to confirm that the efficacy/effectiveness can be seen not exclusively under ideal clinical conditions.

Heterogeneity was moderate in RCTs and considerable in time-trend analyses. In the study of Yoshikawa and co-workers only one country was covered in contrast to the other RCTs, in which several countries were involved. In addition, the follow-up period was much shorter in the Japanese study, which could explain the moderate heterogeneity among RCTs. Moreover, the ecological studies were performed in different countries, and the difference in vaccination coverage, sexual behaviour, length of pre- and post-vaccination periods, or source of study data could also be a cause of heterogeneity.

Our findings are consistent with previous reviews and meta-analyses, where it was also found that the prevalence of GW decreased significantly in vaccinated girls [6, 35, 36], and the quadrivalent prophylactic vaccination could prevent HPV infection in men and women [37]. However, high heterogeneity was found in the case of time trend ecological studies, thus, our results should be interpreted with caution.

Gardasil® has been the vaccine of choice worldwide. It has been chosen by health authorities in the United States, Australia, New Zealand, Canada, Switzerland, Italy, Spain, and Sweden for regional or national vaccination programmes against cervical cancer. The United Kingdom substituted the bivalent vaccine with the quadrivalent one in 2012, since the government clarified that the aim is to protect girls against the types of HPV that cause cervical cancer and those that cause GW [38]. A study from Australia shows that cases of GW have nearly disappeared since 2007, when the national vaccination programme against cervical cancer using Gardasil® was introduced [31]. In New-Zealand the relative risk of GW diagnosis decreased after the introduction of the quadrivalent HPV vaccination in late 2008 [39]. In Spain, there was a decline in GW when female subjects were vaccinated with quadrivalent HPV vaccine, although the bivalent vaccine showed no efficacy against GW [40].

To the best of our knowledge, our work is the first meta-analysis related to GW which is based on the results of both RCTs and time-trend analyses, and carried out a combined analysis in men and women. The advantage of this paper is the high number of participants involved in ecological studies and consequently in this meta-analysis.

However, there are some limitations of this study. First, particularly, the per-protocol population in RCTs is not entirely representative of the general population of women because of the exclusion and inclusion criteria of the trials (for example, low lifetime number of sex partners, no past history of abnormal Pap test or external genital abnormality). Therefore, our results cannot be generalized to other populations with different background risks. Second, in RCTs, many participants from a lot of countries were involved. We tried to come into contact with several authors to get raw data, but the response rate was very low. Thus we cannot state that the actual subsets involved in the different included studies differed totally, but it may not entail extreme distortions. Third, we had to exclude some reports, because we were unable to extract the necessary information from them (mainly the absolute number of genital warts in the pre- and postvaccination periods). Fourth, since our aim was to evaluate the population-level effect of the vaccine, we included time-trend analyses despite the high risk of bias. In addition, this meta-analysis covers time-trend analyses mainly from Australia (1 from England, 1 from the USA, 1 from Belgium and 5 from Australia). Fifth, those articles in which HPV vaccine was administered in the framework of private care, and it was not covered by the National Health Insurance, were excluded, thus the present analysis does not take into account the effect of vaccines that were purchased. We think, that the persons who purchased the HPV vaccine may have different socioeconomic status and sexual habits than those people who were vaccinated in the framework of national/school-based programme, therefore the inclusion of privately vaccinated persons may cause a distortion on the outcome of the study.

## Conclusions

The results from RCTs and time-trend analyses – representing more than 13,000 000 participants – have shown that the quadrivalent HPV vaccine is highly effective in preventing HPV 6/11 related GW both in women and men which gives an additional value to the application of this type of vaccine. Our meta-analysis provides up-to-date information for the public about the effectiveness of HPV vaccination. Teenagers and their parents should acquire better knowledge about HPV infection and prevention. This is of very high importance, because rumours about vaccine safety have been one of the principal obstacles for the acceptance of HPV vaccination by the public. Despite the early implementation of national vaccination programs, in the majority of developed countries coverage rates remain unsatisfactory [41].

Furthermore, the present work provides reliable information for obstetrician–gynaecologists and other health care providers who should raise the attention of parents and patients for the benefits of HPV vaccination and offer HPV vaccines. Additionally, our results demonstrating strong evidence of quadrivalent HPV vaccine effectiveness can help the governments for making decisions about the implementation of the vaccination. It would be recommended to include the quadrivalent HPV vaccine in routine immunization programme because of its high effectiveness not only against cancer but also against GW.

In summary, our results clearly show that the ecological impact of the quadrivalent HPV vaccine is high and its introduction in many countries is strongly suggested.

## Supplementary information

**Additional file 1 **Graphical output from Copas analysis of 8 RCTs (**A**) and 8 ecological studies (**B**) (a): Funnel plot; (b): contour plot; (c): treatment effect plot; (d): P-value plot.

## Data Availability

Not applicable.

## Abbreviations

HPV: human papillomavirus
GW: genital wart
RCT: randomised controlled trial
FDA: Food and Drug Administration
WHO: World Health Organization
OR: odds ratio
CI: confidence interval
PRISMA: Preferred Reporting Items for Systematic Reviews and Meta-Analyses
PICO: patient, intervention, comparison, outcome
PROSPERO: International Prospective Register of Systematic Reviews
GRADE: Grading of Recommendations, Assessment, Development and Evaluation
USA: United States of America
