# Supplementary material for: The quadrivalent HPV vaccine is protective against genital warts: a meta-analysis
Source: BMC Public Health. 2020 May 28;20:691. doi: 10.1186/s12889-020-08753-y (PMC7254696; doi:10.1186/s12889-020-08753-y)

Graphical output from Copas analysis of 8 RCTs (**A**) and 8 ecological studies (**B**)

(a): Funnel plot; (b): contour plot; (c): treatment effect plot; (d): P-value plot

**A**


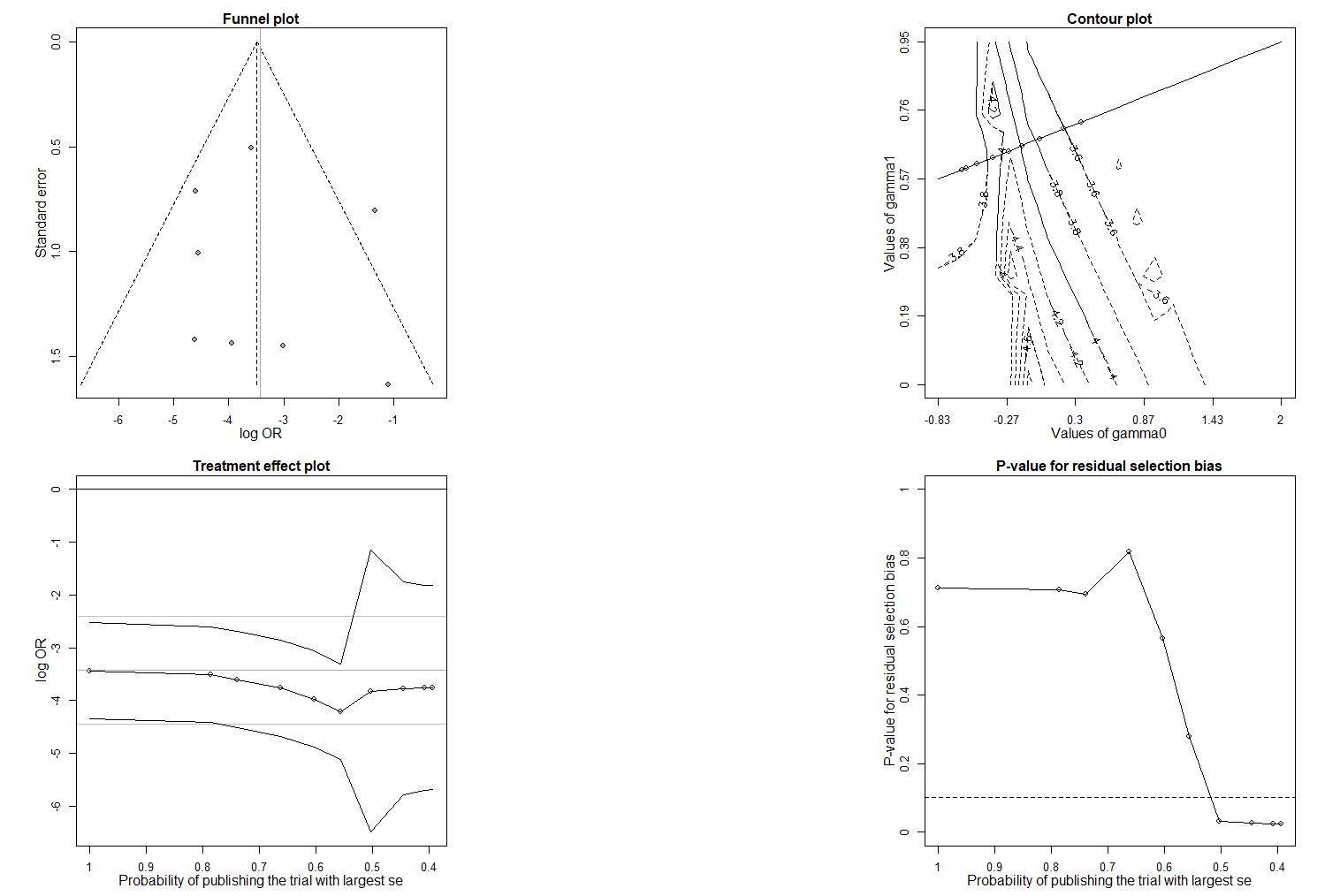


**B**


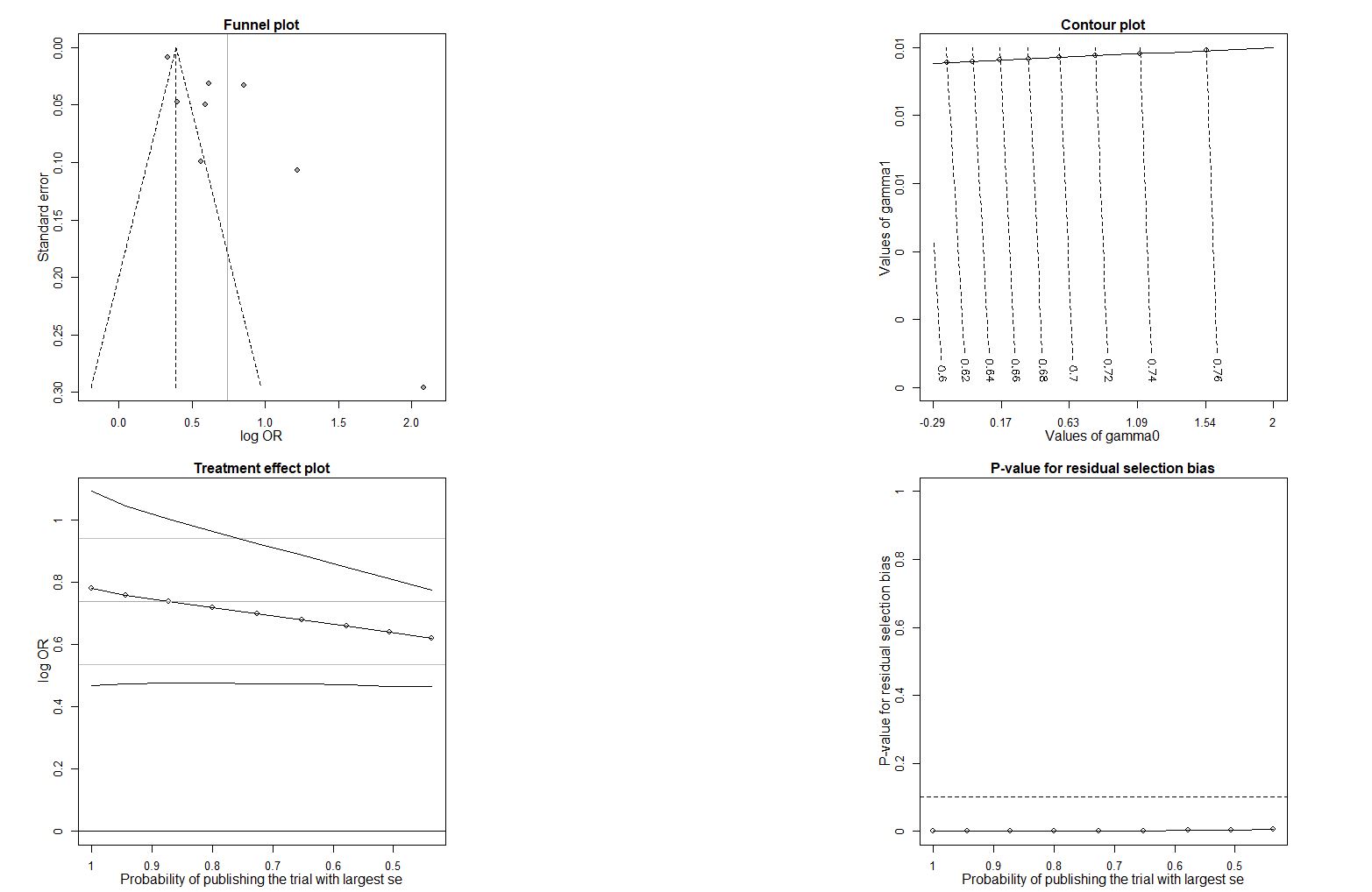

Supplement: Supplementary file 1 — Additional file 1 Graphical output from Copas analysis of 8 RCTs (A) and 8 ecological studies (B) (a): Funnel plot; (b): contour plot; (c): treatment effect plot; (d): P-value plot. [file 12889_2020_8753_MOESM1_ESM.docx]
